# WISP-3 facilitates CCL4-dependent monocyte migration and M1 polarization in rheumatoid arthritis by inhibiting miR-6894-5p

**DOI:** 10.7150/ijms.122642

**Published:** 2026-01-01

**Authors:** Guo-Shou Wang, Kun-Tsan Lee, Syuan-Ling Lin, Sheng-Mou Hou, Yi-Chin Fong, Chih-Hsin Tang, Chih-Yang Lin

**Affiliations:** 1 Department of Medicine, MacKay Medical University, New Taipei City, Taiwan.; 2 Department of Orthopedic Surgery, MacKay Memorial Hospital, Taipei, Taiwan.; 3 Department of Post-Baccalaureate Medicine, National Chung-Hsing University, Taichung, Taiwan.; 4 Department of Orthopedics, Taichung Veterans General Hospital, Taichung, Taiwan.; 5 Translational Medicine Research Center, China Medical University Hospital, Taichung, Taiwan.; 6 Department of Research, Taiwan Blood Services Foundation, Taipei, Taiwan.; 7 The Director's Office, Shin Kong Wu Ho-Su Memorial Hospital, Taipei, Taiwan.; 8 Department of Sports Medicine, College of Health Care, China Medical University, Taichung, Taiwan.; 9 Department of Orthopedic Surgery, China Medical University Hospital, Taichung, Taiwan.; 10 Department of Orthopedic Surgery, China Medical University Beigang Hospital, Yunlin, Taiwan.; 11 Department of Pharmacology, School of Medicine, China Medical University, Taichung, Taiwan.; 12 Department of Medical Laboratory Science and Biotechnology, Asia University, Taichung, Taiwan.; 13 Chinese Medicine Research Center, China Medical University, Taichung, Taiwan.; 14 Translational Medicine Center, Shin-Kong Wu Ho-Su Memorial Hospital, Taipei, Taiwan.

**Keywords:** rheumatoid arthritis, WISP-3, CCL4, Monocyte adhesion, M1 polarization

## Abstract

Chronic systemic inflammation and autoimmunity are hallmarks of rheumatoid arthritis (RA), an inflammatory illness that progressively deteriorates joints, resulting in permanent disability. Monocyte adhesion and infiltration into synovial tissue are critical steps in RA progression. WISP-3 (referred to as CCN6) is a member of the CCN family and plays a role in regulating various developmental processes. However, the role of WISP-3 in monocyte adhesion and macrophage polarization in RA remains unknown. Our high-throughput cytokine array data indicate that WISP-3 induces CCL4 synthesis in RA synovial fibroblasts (RASFs), subsequently enhancing monocyte adhesion to the synovium. Result from the GEO database confirm that levels of WISP-3 and CCL4 are markedly higher in RA patients compared to healthy controls. We also elucidated a detailed mechanism by which WISP-3 increases CCL4 synthesis in RASFs and promotes monocyte adhesion to the synovium by reducing miR-6894-5p expression via the MEK and ERK pathways. Furthermore, WISP-3 augments M1 macrophage polarization in the RA microenvironment. Thus, the WISP-3/CCL4 axis may serve as a novel therapeutic goal for RA treatment.

## Introduction

Chronic systemic inflammation and autoimmunity are hallmarks of rheumatoid arthritis (RA), an inflammatory disease that progressively damages the joints and ultimately leads to permanent disability 1, 2. Both bone and cartilage are affected by the abnormal proliferation of synovial cells, driven by complex interactions among various cytokines and cell types involved in the pathogenesis of RA 3, 4. The imbalance between pro- and anti-inflammatory mediators contributes to persistent inflammation, autoimmune dysfunction, and joint destruction 5, 6. Through the production of chondrolytic enzymes and pro-inflammatory cytokines, rheumatoid arthritis synovial fibroblasts (RASFs) play a central role in initiating and sustaining joint degeneration 7, 8. Therefore, the pathological state of the synovium should be a critical focus in the development of therapeutic strategies for RA.

Mononuclear cells play an essential role in the progression of RA. Monocytes and macrophages, key phagocytic cells of the innate immune system, are widely distributed throughout various tissues, including joints 9, 10. They are involved in maintaining tissue homeostasis and protecting against infections 11. Moreover, these cells interact closely with the adaptive immune system, particularly through their secretory mediators in response to T cell activation 12. The pathogenesis of RA is critically dependent on the recruitment of monocytes into the joints and their subsequent adhesion to the synovial membrane 12. Upon migrating to synovial tissues, monocytes differentiate into macrophages and can be polarized into either the M1 or M2 phenotype in response to different environmental stimuli 13. In RA, pro-inflammatory cytokines such as IL-1β and TNF-α induce M1 polarization, leading to elevated levels of inflammatory mediators, enhanced cartilage degradation, and increased osteoclast formation 13-15. M1 macrophages release various pro-inflammatory cytokines that drive the chronic inflammatory response, tissue destruction, and pain characteristic of RA 16. In contrast, M2 macrophages exert anti-inflammatory and tissue-repairing functions. Patients with RA exhibit accelerated monocyte turnover, shortened circulation time, and a pronounced tendency for monocyte accumulation in the joints 17. After migrating into the synovium, these monocytes may polarize into M1 macrophages that promote inflammation or into M2 macrophages that contribute to inflammation resolution. Notably, the M1/M2 ratio in the synovial tissue of RA patients is significantly higher than that observed in healthy individuals 18. Consequently, therapeutic strategies aimed at inhibiting M1 polarization or restoring the M1/M2 balance may hold potential in mitigating joint inflammation and tissue damage in RA.

In addition to cellular dysregulation, the pathogenesis of RA is profoundly influenced by disturbances in gene regulatory mechanisms, particularly those mediated by microRNAs (miRNAs) 19. miRNAs are small, non-coding RNA molecules of approximately 22 nucleotides that play essential roles in post-transcriptional regulation of gene expression and are evolutionarily conserved components of the cellular regulatory network. They exhibit dynamic and tightly controlled expression patterns, enabling cells to respond precisely to environmental stimuli 20. Several signaling pathways, including the p53, JAK-STAT, AP-1, MAPK, Toll-like receptor (TLR), and NF-κB pathways, regulate miRNA synthesis and are themselves modulated by miRNA feedback mechanisms, thereby sustaining the chronic inflammatory milieu characteristic of RA 21, 22. Alterations in these pathways affect immune cell activation and cytokine production, thereby amplifying synovial inflammation. Notably, miRNA expression varies substantially across different macrophage polarization states, allowing macrophages to adapt to inflammatory or anti-inflammatory cues. Dysregulation of specific miRNAs, such as miR-155 and miR-146a, has been shown to disrupt monocyte and macrophage function, contributing to the persistence of inflammation and joint destruction in RA 19, 23. Therefore, elucidating the specific miRNAs and their upstream regulatory factors involved in macrophage activation may provide new insights into the molecular mechanisms underlying RA and identify potential therapeutic targets.

Six matricellular proteins make up the CCN family, and they play crucial roles in the primary pathophysiological functions of inflammation, migration and angiogenesis 24. WISP-3 (referred to as CCN6) is part of the CCN family and plays a role in regulating various developmental effects 25. In RA synovium, WISP-3 mRNA expression is markedly elevated compared with osteoarthritis and normal synovial tissue, and its transcription can be further enhanced by pro-inflammatory cytokines in RASFs 26. In cartilage, WISP-3 contributes to extracellular matrix homeostasis and exerts complex, dose- and context-specific regulation of matrix metalloproteinases such as ADAMTS-5 and MMP-10 27. These findings underscore the context-specific nature of WISP-3: in chondrocytes and cartilage, it helps maintain matrix integrity, whereas in RA FLS, as demonstrated in the present study, it promotes chemokine expression and monocyte adhesion within an inflammatory microenvironment. It is noteworthy that the levels of WISP-3 protein in RA and normal synovium are comparable, indicating that there may not be a coordinated regulation between WISP-3 protein and mRNA 26. Notably, WISP-3 seems to play a significant part in the progression of juvenile idiopathic arthritis, which encompasses chronic inflammatory arthropathies occurring in childhood 28. However, the role of WISP-3 in monocyte adhesion to the synovium and polarization during RA remains unknown. Our high-throughput cytokine array data indicate that WISP-3 induces CCL4 production in RASFs, subsequently enhancing monocyte adhesion. Inhibition of miR-6894-5p expression through the MEK and ERK pathways is participated in WISP-3-regulated effects. Interestingly, conditioned medium (CM) from WISP-3-stimulated RASFs augments M1 polarization. Thus, the WISP-3/CCL4 axis represents a novel therapeutic goal for RA treatment.

## Material and Methods

### Materials

Antibodies against MEK-1 (H-8) (Catalog No: SC-6250) and ERK (D-2) (Catalog No: SC-1647) were sourced from Santa Cruz Biotechnology (CA, USA). Cell Signaling Technology (Danvers, MA, USA) supplied the p-MEK1/2 (Ser221) (166F8) (Catalog No: 2338) and p-p44/42 MAPK (Erk1/2) (Thr202/Tyr204) (Catalog No: 4370) antibodies. CCL4 (GTX17201) antibodie was purchased from GeneTex (Hsinchu, Taiwan). β-actin antibody (Catalog No: a5441) was obtained from Sigma-Aldrich (St. Louis, MO, USA). Recombinant human WISP-3 was obtained from PerpoTech, located in Rocky Hill, NJ, USA. All other chemicals used in this study were sourced from Sigma-Aldrich (St. Louis, MO, USA).

### Cell culture

Human RASFs cell line (MH7A) was acquired from RIKEN (Ibaraki, Japan). Human monocyte cell line THP-1 was purchased from the American Type Culture Collection (Manassas, VA, USA). Cells were cultured in RPMI-1640 medium containing 10% FBS, penicillin, and streptomycin at 37°C under a humidified atmosphere of 5% CO_2_ 29. All experiments were performed with mycoplasma-free cells.

### Investigation of the adhesion of monocytes to RASFs

MH7A cells (1 × 10^5^) were seeded in 12-well plates and cultured until they reached approximately 90% confluence. Cells were then stimulated with recombinant WISP-3 (100 ng/mL) for 24 h. In parallel, human monocytes were labeled with BCECF-AM (10 μM; Thermo Fisher Scientific, Inc.) for 1 hour at 37°C according to the manufacturer's instructions. After labeling, monocytes were washed, resuspended in fresh medium, and monocytes (1 × 10^5^ cells per well) were subsequently co-cultured with WISP-3-treated MH7A cells for 1 h at 37 °C. Non-adherent monocytes were removed by gentle washing with PBS, while adherent monocytes were visualized and quantified using a fluorescence microscope. Five randomly selected fields per well were analyzed using ImageJ for fluorescence intensity and cell counts 30.

### Human chemokine antibody array

Secreted chemokines were analyzed using the Human Chemokine Antibody Array C1 (RayBiotech, Inc., Norcross, GA, USA; Cat. No. AAH-CHE-1). MH7A cells were cultured under standard conditions until ~70-80% confluence. To reduce background signals, cells were serum-starved in medium containing 0% FBS for 12-16 h before stimulation. Cells were then treated with recombinant WISP-3 (100 ng/mL) for 24 h, and the protein was collected. The experiment was performed according to the manufacturer's protocol. Briefly, array membranes pre-coated with capture antibodies were blocked with the supplied blocking buffer, then incubated with 1 mL of each sample (cell lysis). After washing, membranes were incubated with the Biotinylated Antibody Cocktail, followed by HRP-conjugated streptavidin. The Fujifilm LAS-3000 imaging equipment was used to view the blot membranes.

### Prediction of miRNAs targeting CCL4

To predict miRNAs that may target CCL4, we used the miRWalk database (http://mirwalk.umm.uni-heidelberg.de/). Candidate predictions were cross-validated with the miRDB database, applying a confidence threshold of ≥0.8. Through this combined approach, we identified five candidate miRNAs with potential binding affinity for CCL4. The primer sequences used for subsequent validation are provided in Table 1.

### Real-time qPCR

As directed by the manufacturer, RNA was isolated from RASFs using a TRIzol kit (MDBio, Taiwan). Using a NanovueTM Spectrophotometer (GE Healthcare, WI, USA), the quantity and quality of the RNA were evaluated based on A260 readings. cDNA synthesis was performed using an M-MLV RT kit (Invitrogen, CA, USA) and 1 μg of total RNA in accordance with the manufacturer's instructions. The KAPA SYBR® FAST qPCR Kit was supplied by Applied Biosystems, CA, USA, to perform real-time qPCR 31, 32. Primer sequences were designed using a PrimerBank, and the detailed sequences are listed in Table 1.

### Western blot analysis

The extracted proteins (30 μg) were resolved using SDS-PAGE, and PVDF membranes were then transferred in accordance with the protocols described in our previous publications 33, 34. After blocking the membranes for an hour in PBST containing 4% non-fat milk, they were treated with primary antibodies and secondary antibodies conjugated with HRP for an additional hour. A computer densitometer equipped with an ImageQuant LAS4000 (GE Healthcare Life Sciences) was used to measure the amount of protein.

### Bioinformatics analysis

Publicly available transcriptome data were obtained from the Gene Expression Omnibus (GEO) database (accession number GSE77298). This dataset includes synovial biopsy specimens from patients with end-stage rheumatoid arthritis (RA; n = 16) and from individuals without joint disease (healthy controls, HC; n = 7). In particular, the expression levels of WISP-3 (WNT1-inducible signaling pathway protein 3), CCL4, CCL5, CD14, CD68, TNFα, iNOS, CD86, Arg-1, and CD204 were extracted from the dataset for focused evaluation. The differential expression profiles of these genes were visualized using boxplots generated in GraphPad Prism 8.0 software to illustrate their relative enrichment in RA compared with HC 35.

### Cell transfection

Small interfering RNAs (siRNAs) targeting CCL4, MEK, ERK, and control siRNA were synthesized by Santa Cruz Biotechnology (Santa Cruz, USA). Control mimic, miR-29b-3p mimic, and Lipofectamine 2000 were obtained from Invitrogen (Carlsbad, CA, USA). Transient transfection of siRNA or miRNA mimic was performed using Lipofectamine 2000 in accordance with the manufacturer's protocol. MH7A cells were seeded in a 6-well plate (5 × 10⁵ cells per well) and cultured for 16 h until reaching 80% confluence. One hour before transfection, the culture medium was replaced with serum- and an-tibiotic-free medium. Cells were incubated with transfection mixtures containing 100 nM silencing siRNA, control siRNA, miR-6894-5p, or control mimic for 24 h. Cells were harvested for further analysis by qPCR or Western blot to assess knockdown efficiency and downstream effects 36.

### THP-1 differentiation and macrophage polarization

THP-1 monocytes were seeded at a density of 1 × 10^6^ cells per mL per well in 6-well plates. To induce differentiation into M0 macrophages, cells were treated with 200 nM phorbol 12-myristate 13-acetate (PMA) for 24 hours under standard culture conditions (37°C, 5% CO₂). Following this period, cells ceased proliferation and adopted a macrophage-like phenotype (M0). Subsequently, M0 macrophages were further stimulated for an additional 24 hours with CM derived from WISP-3-treated MH7A cultures. After polarization treatment, total RNA was extracted, and the expression of M1 (e.g., TNF-α, iNOS, CD86) and M2 (e.g., Arg-1, CD204) macrophage markers was quantified by qPCR.

### Statistical analysis

Quantified data were analyzed using GraphPad Prism 8.0 software. All values are presented as the mean ± standard deviation (SD). Statistical significance between experiment groups was evaluated using the Student's t-test to compare two unpaired groups. *p*<0.05 was considered to indicate a statistically significant difference.

## Results

### WISP-3 promotes CCL4 production in RASFs and facilitates monocyte adhesion

**Figure 1:**
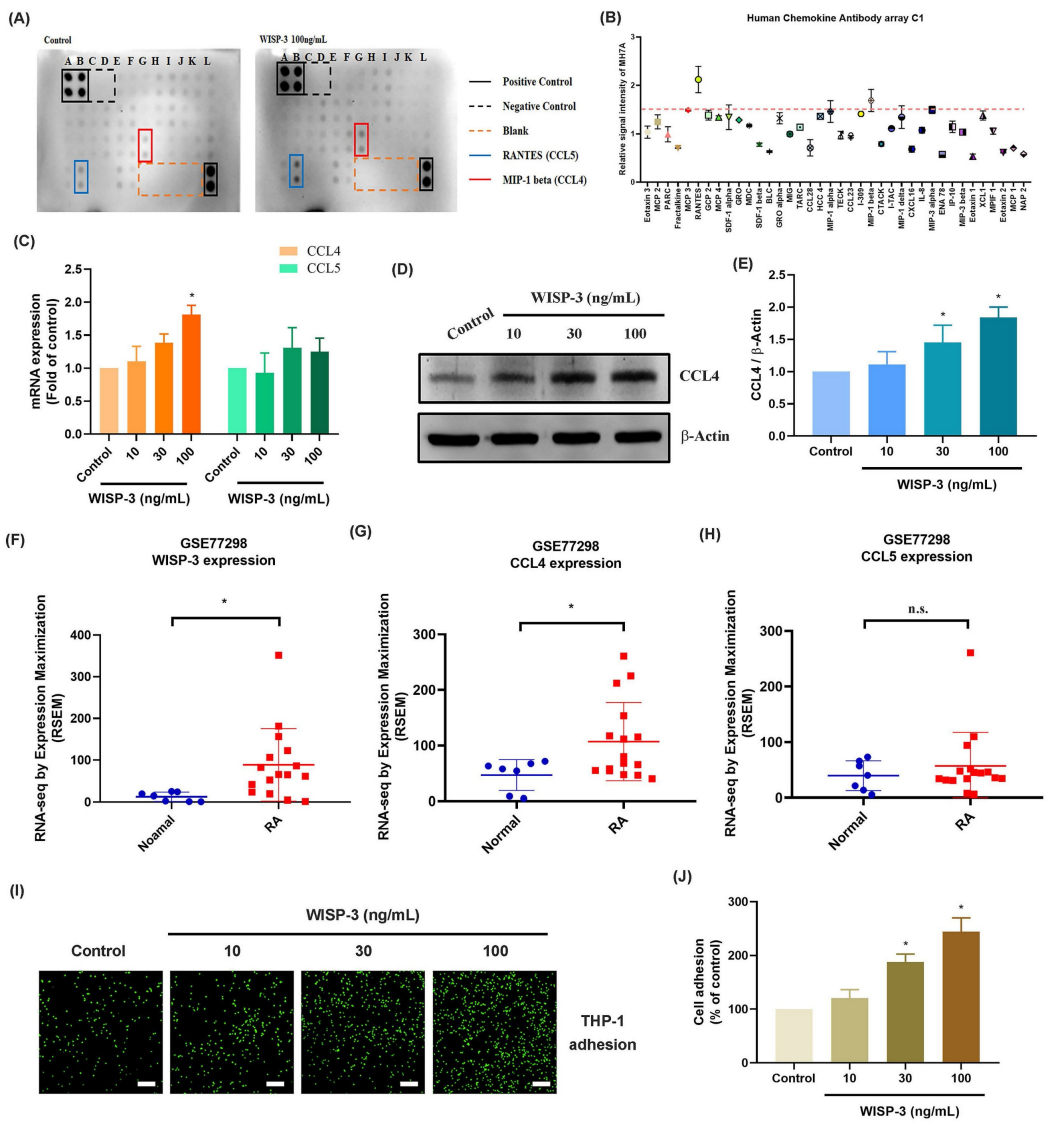
** WISP-3 promotes CCL4 production in RASFs and enhances monocyte adhesion.** (A&B) Results of cytokine array showing the expression of inflammatory factors by RASFs treated with WISP-3 for 24 h. (C-E) RASFs were treated with WISP-3 for 24 h, the CCL4 expression was examined by qPCR and Western blotting. (F-H) The levels of WISP-3, CCL4 and CCL5 determined using the GSE77298 dataset. (I&J) Fluorescence microscope images of THP-1 cells adhered to RASFs, following incubation with WISP-3 for 24 h. (K&L) Representative fluorescence images and quantitative analysis of THP-1 monocyte adhesion to RASFs treated with WISP-3 (100 ng/mL). Scale bar = 100 µm. All experiments were repeated 3 to 5 times. * *p* < 0.05 compared with the control group.

WISP-3 has a reported function in cartilage growth and maintenance during arthritis 27. We therefore investigated the functions of WISP-3 in cytokine production in RASFs. High-throughput cytokine array data indicated that WISP-3 most strongly upregulated CCL4 and CCL5 expression (Fig. 1A&B). However, RASFs stimulated with WISP-3 markedly enhanced CCL4, but not CCL5, mRNA expression in a concentration-dependent manner (Fig. 1C). Additionally, WISP-3 increased CCL4 protein production in RASFs (Fig. 1D&E). Data from the GEO database also confirm that WISP-3 and CCL4, but not CCL5, are elevated in RA patients compared to healthy controls (Fig. 1F-H). Interestingly, stimulation of RASFs with WISP-3 promoted monocyte adhesion to RASFs (Fig. 1I&J), whereas CCL4 siRNA significantly inhibited WISP-3-induced monocyte adhesion (Fig. 1K&L). Thus, WISP-3 induces CCL4 synthesis in RASFs, which in turn promotes monocyte adhesion.

### WISP-3 promotes CCL4 production in RASFs and subsequently facilitates monocyte adhesion by reducing miR-6894-5p via the MEK and ERK pathways

**Figure 2:**
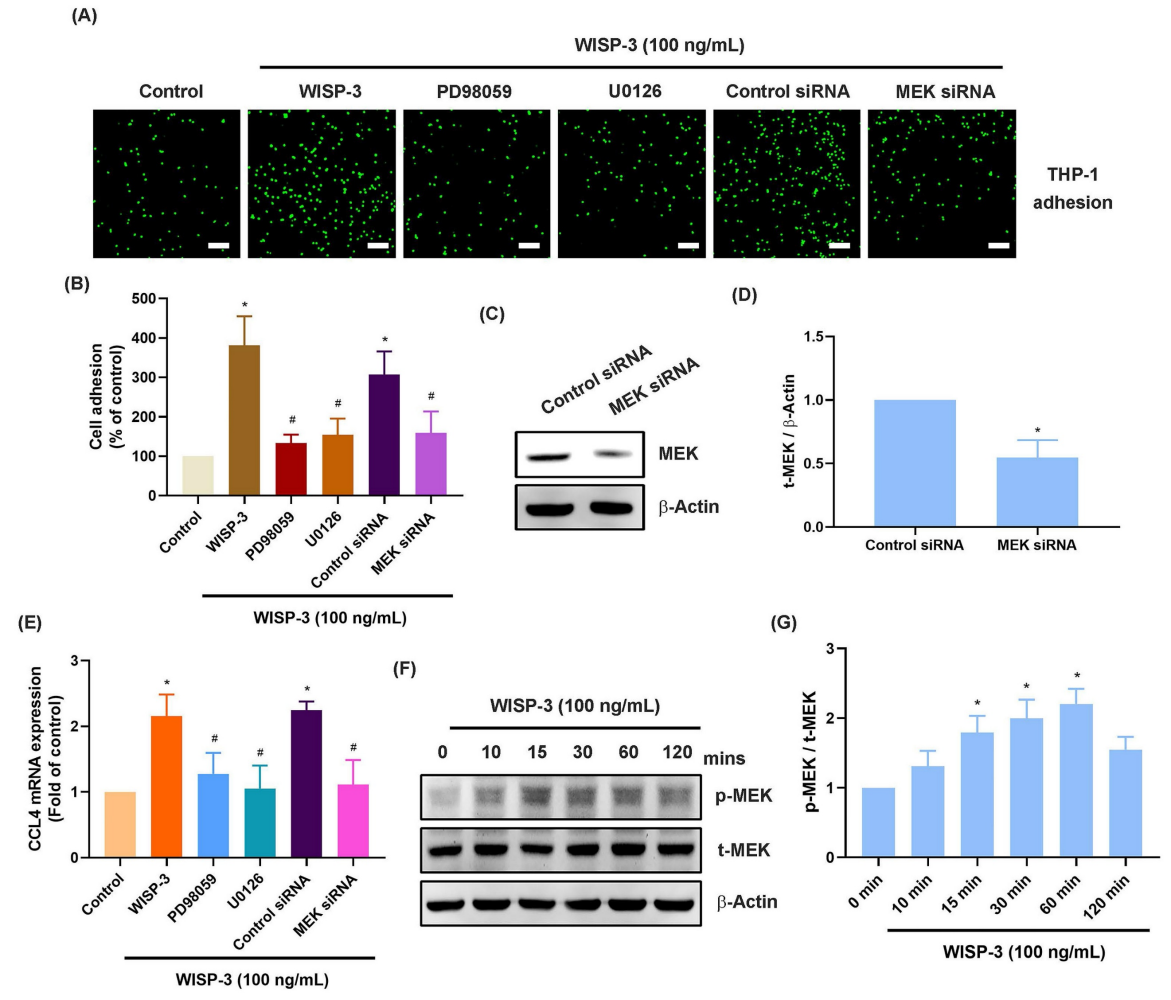
** MEK is involved in WISP-3-induced CCL4 production and monocyte adhesion.** (A-E) THP-1 adhesion and CCL4 expression in RASFs incubated with MEK inhibitors (PD98059 and U0126) or transfected with MEK siRNA and then stimulated with WISP-3 for 24 h. (C&D) RASFs were transfected with MEK siRNA, the MEK expression was examined by Western blotting. (F&G) RASFs were treated with WISP-3, the MEK phosphorylation was examined by Western blotting. Scale bar = 100 µm. All experiments were repeated 3 to 5 times. * *p* < 0.05 compared with the control group. # *p* < 0.05 compared with the WISP-3-treated group.

**Figure 3:**
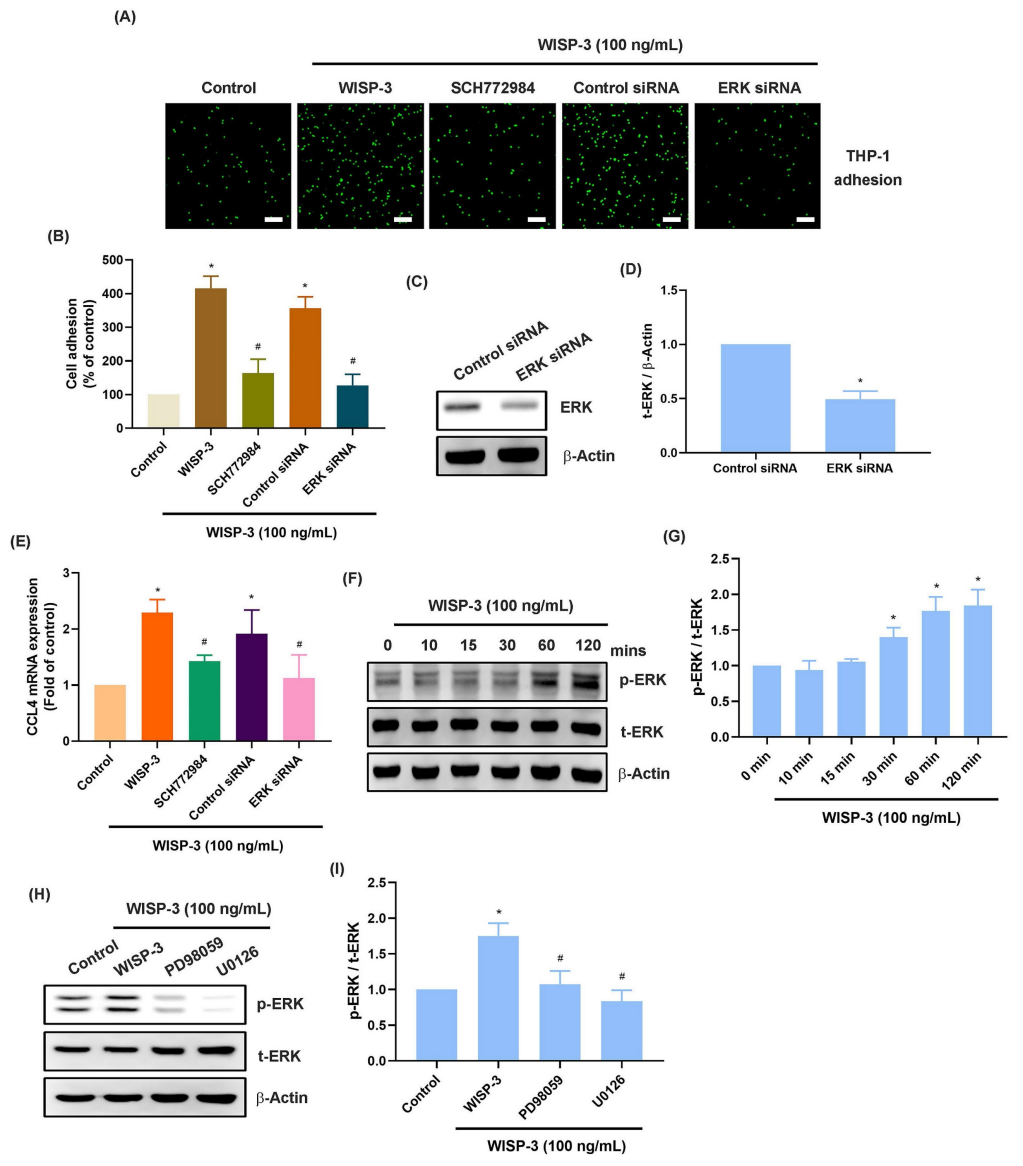
** ERK is involved in WISP-3-induced CCL4 production and monocyte adhesion.** (A-E) THP-1 adhesion and CCL4 expression in RASFs incubated with ERK inhibitor (SCH772984) or transfected with ERK siRNA and then stimulated with WISP-3 for 24 h. (C&D) RASFs were transfected with ERK siRNA, the ERK expression was examined by Western blotting. (F&G) RASFs were treated with WISP-3, the MEK phosphorylation was examined by Western blotting. (H&I) RASFs were treated with MEK inhibitors then stimulated with WISP-3, the ERK phosphorylation was examined by Western blotting. Scale bar = 100 µm. All experiments were repeated 3 to 5 times. * *p* < 0.05 compared with the control group. # *p* < 0.05 compared with the WISP-3-treated group.

The MEK and ERK signaling cascades are crucial for monocyte adhesion and infiltration during arthritis 37, 38. Treatment of RASFs with MEK inhibitors (PD98059 and U0126) abolished WISP-3-enhanced promotion of monocyte adhesion and CCL4 production (Fig. 2A-E). Transfection of RASFs with MEK siRNA produced similar results (Fig. 2A-E). CCL4 administration increased MEK phosphorylation in RASFs (Fig. 2F&G). Furthermore, the ERK inhibitor (SCH772984) and ERK siRNA blocked WISP-3-enhanced monocyte adhesion and CCL4 expression (Fig. 3A-E). CCL4 stimulation also augmented ERK phosphorylation, which was reversed by application with MEK inhibitors (Fig. 3F-I), indicating that the MEK/ERK signaling pathway controls WISP-3-mediated CCL4 synthesis and monocyte adhesion to RASFs.

**Figure 4:**
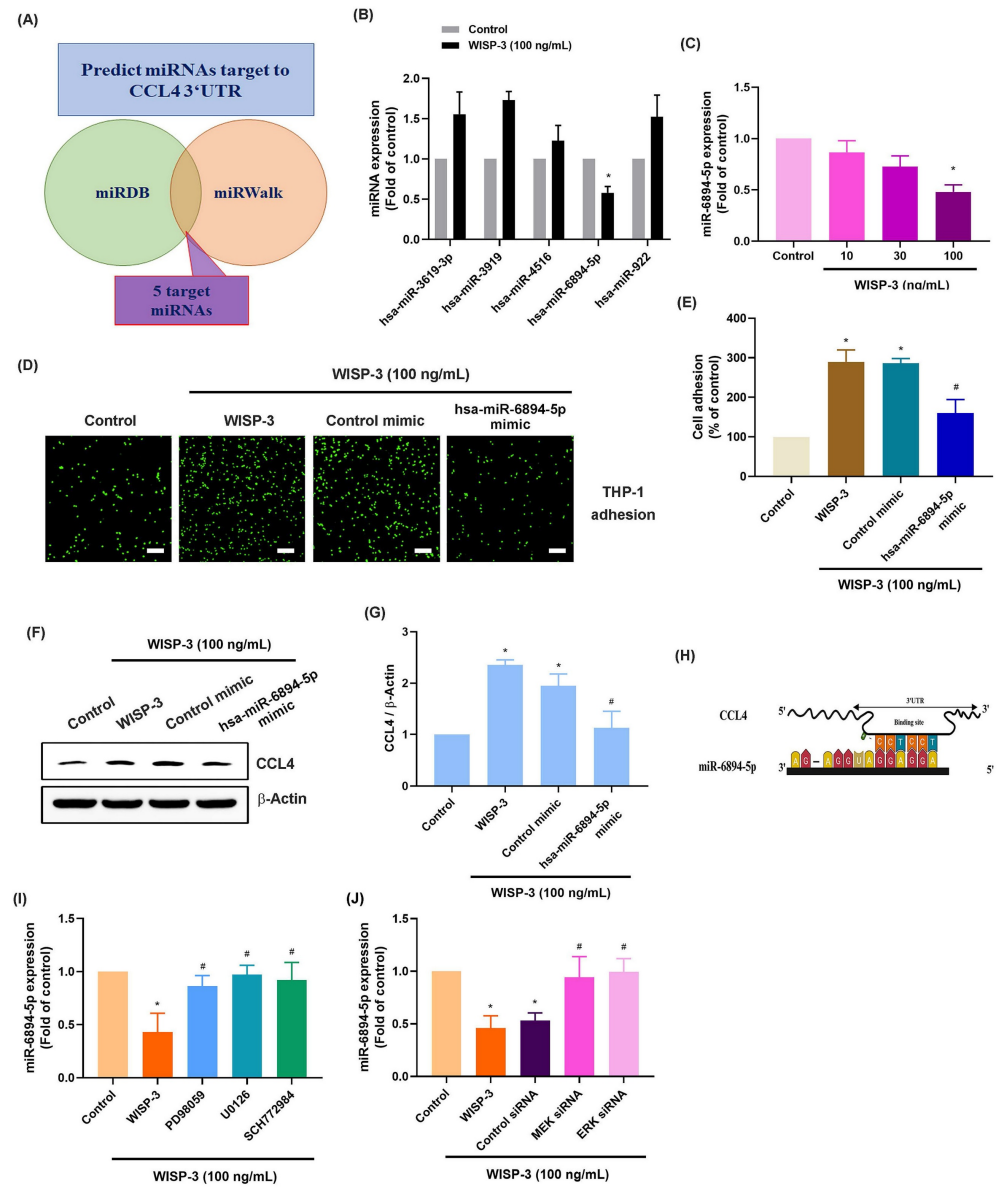
** miR-6894-5p controls WISP-3-induced CCL4 production and monocyte adhesion.** (A) The diagrams illustrate the selection of miRNA candidates aimed at CCL4. (B&C) RASFs were treated with WISP-3 for 24 h, the indicated miRNA expression was examined by qPCR. (D-G) THP-1 adhesion and CCL4 expression in RASFs transfected with miR-6894-5p mimic and then stimulated with WISP-3 for 24 h. (H) Schematic illustration of the predicted binding site for miR-6894-5p within the 3′-UTR of CCL4 mRNA, showing the complementary base-pairing interaction. (I&J) RASFs were treated with MEK and ERK inhibitors or siRNA then stimulated with WISP-3 for 24 h, the miR-6894-5p expression was examined by qPCR. Scale bar = 100 µm. All experiments were repeated 3 to 5 times. * *p* < 0.05 compared with the control group. # *p* < 0.05 compared with the WISP-3-treated group.

miRNAs are small non-coding RNAs that control different pathological functions in RA 39, 40. Employing two bioinformatics tools (miRDB and miRWalk), we discovered five miRNAs that directly interact with the 3' UTRs of CCL4 (Fig. 4A). WISP-3 treatment primarily diminished miR-6894-5p expression among them (Fig. 4B). RASFs incubated with WISP-3 showed a reduction in miR-6894-5p expression that was dependent on the concentration (Fig. 4C). Further experiments were conducted to determine whether WISP-3 promotes monocyte adhesion by regulating miR-6894-5p. To test this, RASFs were transfected with a miR-6894-5p mimic, which effectively reversed the WISP-3-induced increases in monocyte adhesion and CCL4 production (Fig. 4D-G). Consistent with these findings, bioinformatic analysis predicted a direct binding site for miR-6894-5p within the 3′ UTR of CCL4 mRNA (Fig. 4H), suggesting that CCL4 is a potential downstream target of miR-6894-5p. Moreover, both pharmacological inhibition (MEK and ERK inhibitors) and siRNA-mediated knockdown of MEK and ERK during incubation reversed the effects induced by WISP-3 on miR-6894-5p production (Fig. 4I-J). This indicates that WISP-3 enhances CCL4 synthesis and monocyte adhesion by reducing miR-6894-5p through the MEK and ERK pathway.

### WISP-3-treated RASFs enhance M1 macrophage polarization

**Figure 5:**
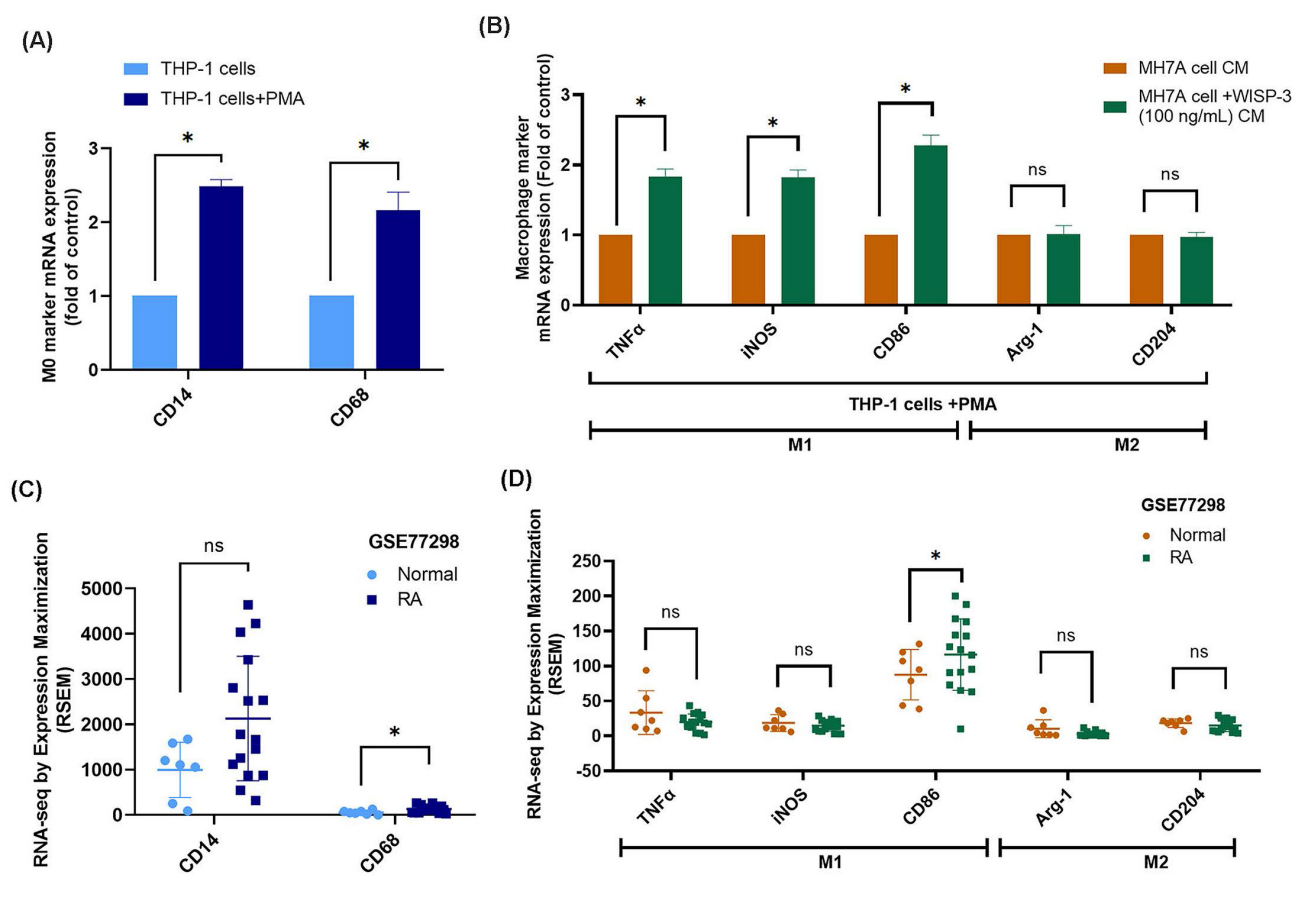
** WISP-3 enhances M1 macrophage polarization.** (A) qPCR analysis of THP-1 cells after a 24 h incubation with PMA. (B) mRNA expression analysis via qPCR following a 24 h treatment of RASFs with WISP-3 and subsequent application of conditioned medium to M0 macrophages. (C&D) Analysis of RNA-seq data from the GEO dataset GSE77298 showing expression levels of macrophage markers in synovial tissues from normal and RA patients. All experiments were repeated 3 to 5 times. * *p* < 0.05 compared with the control group.

Migrated monocytes/macrophages in the synovial membrane polarize into either the M1 or M2 phenotype during RA development 41. Upon stimulation with PMA, the mRNA expression of M0 markers CD14 and CD68 was significantly increased, confirming the differentiation of THP-1 cells into M0 macrophages (Fig. 5A). When these M0 macrophages were exposed to CM from WISP-3-stimulated RASFs, their polarization shifted toward the M1 phenotype, as evidenced by increased expression of TNF-α, iNOS, and CD86, whereas M2 markers (Arg-1, CD204) remained unchanged (Fig. 5B). Subsequently, analysis of the GSE77298 dataset showed that CD68 expression was significantly elevated in RA synovial tissues compared with normal controls, while CD14 levels were not significantly different (Fig. 5C). Moreover, the M1 marker CD86 was markedly upregulated in RA samples, whereas TNF-α, iNOS, and the M2 markers Arg-1 and CD204 displayed no significant alterations (Fig. 5D). These findings collectively indicate that WISP-3 promotes macrophage polarization toward a pro-inflammatory M1 phenotype, which may contribute to the inflammatory microenvironment of RA.

## Discussion

RA's autoimmune inflammatory nature leads to irreversible damage to the affected joints, while the cause of immune system dysfunction remains not fully understood 42. Common features encompass synovial inflammation and the infiltration of immune cells, with activated synovial fibroblasts playing a role in joint inflammation and cartilage destruction through the production of proinflammatory mediators. This contributes to a "vicious cycle" of disease activity within the RA synovial microenvironment 43. We used RASFs as an experimental cell model. A high-throughput cytokine array identified CCL4 as the most potent chemokine induced by WISP-3 stimulation in RASFs. We also demonstrated that WISP-3 promotes CCL4 generation in RASFs and augments monocyte adhesion by diminishing miR-6894-5p via the MEK and ERK pathways. Importantly, CM from WISP-3-treated RASFs promotes macrophage polarization into the M1 phenotype. The WISP-3/CCL4 axis may serve as a potential therapeutic strategy for RA remedy.

Macrophages display extraordinary plasticity, capable of polarizing from a neutral state (M0) to a pro-inflammatory M1 phenotype in response to stimuli like LPS and IFN-γ. Polarization takes on added importance due to the fact that a heightened M1/M2 ratio can exacerbate inflammation and tissue injury, thereby aiding in the advancement of the disease 44, 45. Macrophages' capacity to alternate between these phenotypes highlights their promise as therapeutic targets. M1 macrophage markers are elevated in RA patients compared to healthy individuals 37. Our results indicate that CM from WISP-3-stimulated RASFs promotes M1 macrophage polarization. These data suggest that pro-inflammatory M1 macrophages contribute to WISP-3-mediated RA development.

The MEK and ERK signaling pathways are essential for numerous biological process, including differentiation, proliferation and apoptosis 46, 47. Studies on RA have demonstrated that NGF enhances monocyte adhesion through activation of the TrkA, MEK/ERK, and AP-1 signaling cascades 37. Similarly, nesfatin-1 promotes monocyte migration by upregulating CCL2 expression via the MEK/ERK and p38 signaling pathways 38. These findings highlight the pivotal role of MEK and ERK signaling in the pathogenesis of RA. Consistent with these observations, our data show that pharmacological inhibition of MEK or ERK effectively blocked WISP-3-induced CCL4 expression and monocyte adhesion. Moreover, siRNA-mediated knockdown of MEK and ERK produced comparable effects, further confirming their involvement in this process. In addition, WISP-3 stimulation markedly enhanced the phosphorylation of MEK and ERK. Interestingly, pretreatment with MEK inhibitors suppressed WISP-3-induced ERK phosphorylation, indicating that ERK acts downstream of MEK and participates in WISP-3-mediated CCL4 synthesis and monocyte adhesion.

miRNAs are crucial post-transcriptional regulators that modulate gene expression through complete or partial base pairing with the 3′UTR of target mRNAs, thereby suppressing translation or promoting mRNA degradation 48, 49. Growing evidence suggests that dysregulated miRNA-target interactions contribute to the chronic inflammation and joint destruction characteristic of rheumatoid arthritis (RA), and manipulating these pathways may represent a promising therapeutic strategy 48, 49. For instance, miR-103a-3p mediates TNF-α-induced synovial inflammation and bone erosion by targeting MAP3K7 and DKK1 50, while miR-548aj-3p and miR-3127-3p attenuate RANKL-driven expression of pro-inflammatory cytokines and matrix-degrading enzymes in both osteoarthritis and RA 51. In oncology, circAFF2 acts as a molecular “sponge” that sequesters miR-6894-5p, relieving its repression of ANTXR1 and thereby facilitating gastric cancer progression 52. However, the biological function of miR-6894-5p in RA has not been previously reported. In the present study, we identified miR-6894-5p as a novel downstream target involved in WISP-3-mediated signaling in RA synovial fibroblasts. Bioinformatic prediction and experimental validation revealed that miR-6894-5p directly interacts with the 3′UTR of CCL4 mRNA. WISP-3 stimulation significantly suppressed miR-6894-5p expression, leading to increased CCL4 production and enhanced monocyte adhesion. Conversely, transfection of a miR-6894-5p mimic reversed these effects, confirming the inhibitory role of this miRNA in WISP-3-driven pro-inflammatory responses. Moreover, inhibition of miR-6894-5p by WISP-3 was abolished by blocking the MEK and ERK signaling pathways, indicating that WISP-3 regulates CCL4 synthesis and monocyte adhesion through MEK/ERK-dependent suppression of miR-6894-5p. Together, these findings uncover a novel WISP-3/MEK-ERK/miR-6894-5p/CCL4 axis that contributes to monocyte recruitment and inflammation in RA, providing new insight into the post-transcriptional mechanisms underlying RA pathogenesis and potential targets for therapeutic intervention.

**Figure 6:**
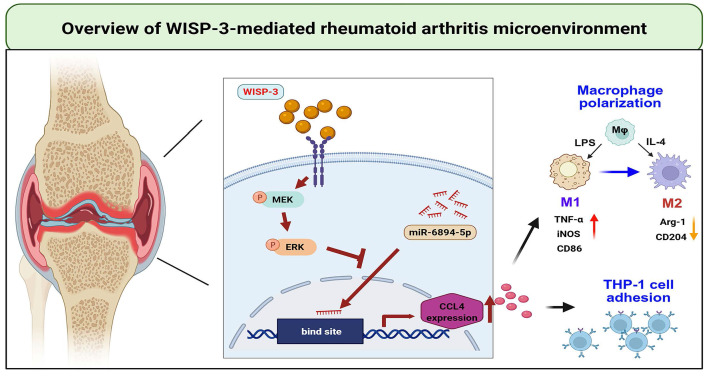
** Schematic diagram illustrating the mechanism underlying the monocyte adhesion and M1 polarization of WISP-3 in RA progression.** WISP-3 increases CCL4 synthesis in RASFs and promotes monocyte adhesion to the synovium by inhibiting miR-6894-5p expression through the MEK and ERK pathways. Furthermore, WISP-3 augments M1 macrophage polarization in the RA microenvironment.

In conclusion, we demonstrate that WISP-3 increases CCL4 synthesis in RASFs and promotes monocyte adhesion to the synovium by inhibiting miR-6894-5p expression through the MEK and ERK pathways. Furthermore, WISP-3 augments M1 macrophage polarization in the RA microenvironment (Fig. 6). Targeting the WISP-3/CCL4 axis may serve as a potential therapeutic strategy for RA treatment.

### Study Limitations

This study has several limitations that should be acknowledged. First, the functional experiments were conducted using immortalized cell lines rather than primary synovial fibroblasts and macrophages from RA patients, which may not fully recapitulate the complexity of the in vivo environment. Second, although our *in vitro* findings (Fig. 5A&B) demonstrated a clear shift of macrophages toward the M1 phenotype upon exposure to WISP-3-stimulated conditioned medium, the transcriptomic analysis of clinical synovial tissues (Fig. 5C&D) showed only partial concordance, with less pronounced differences in M1 marker expression. This discrepancy may reflect heterogeneity among patients, including variations in disease stage, treatment history, or local cytokine milieu, which could influence macrophage polarization in vivo. In early or treated RA, for example, the M1 signature may be less dominant or dynamically regulated. Finally, additional validation using primary RA samples and *in vivo* arthritis models will be necessary to confirm the relevance of the WISP-3/CCL4 axis and its association with macrophage polarization during disease progression.

## Figures and Tables

**Table 1:** List of PCR primer used for the experiments

Target mRNA	Forward primer (5'→3')	Reverse primer (5'→3')
CCL4	CTGTGCTGATCCCAGTGAATC	TCAGTTCAGTTCCAGGTCATACA
CCL5	CCAGCAGTCGTCTTTGTCAC	CTCTGGGTTGGCACACACTT
GAPDH	ACCACAGTCCATGCCATCAC	TCCACCACCCTGTTGCTGTA
		
Target miRNA	Forward primer (5'→3')	
has-miR-922	GCAGCAGAGAATAGGACTACGTC	
has-miR-3619-3p	GGGACCATCCTGCCTGCTGTGG	
has-miR-3919	GCAGAGAACAAAGGACTCAGT	
has-miR-4516	GGGAGAAGGGTCGGGGC	
has-miR-6894-5p	AGGAGGATGGAGAGCTGGGCCAGA	
